# Demonstration of an intense lithium beam for forward-directed pulsed neutron generation

**DOI:** 10.1038/s41598-022-18270-0

**Published:** 2022-08-18

**Authors:** Masahiro Okamura, Shunsuke Ikeda, Takeshi Kanesue, Kazumasa Takahashi, Antonino Cannavó, Giovanni Ceccio, Anastasia Cassisa

**Affiliations:** 1 Brookhaven National Laboratory, Upton, NY 11973 USA; 2 RIKEN, Hirosawa, Wako, Saitama 351-0198 Japan; 3 Tokyo Tech World Research Hub Initiative (WRHI), Tokyo Institute of Technology, 4259 Nagatsuta, Midori-ku, Yokohama, Kanagawa 226-8503 Japan; 4 Nagaoka University of Technology, 1603-1 Kamitomioka, Nagaoka, Niigata 940-2188 Japan; 5 Nuclear Physics Institute of the Czech Academy of Science, Hlavní 130, 250 68 Husinec-Řež, Czech Republic

**Keywords:** Experimental nuclear physics, Radiotherapy, Electrical and electronic engineering, Imaging techniques, Solid-state lasers

## Abstract

As an alternative to research nuclear reactors, a compact accelerator-driven neutron generator that uses a lithium beam driver could be a promising candidate since it produces almost no undesired radiation. However, providing an intense lithium-ion beam has been difficult, and it has been thought that the practical application of such a device would be impossible. The most critical problem of insufficient ion fluxes has been solved by applying a direct plasma injection scheme. In this scheme, a pulsed high-density plasma from a metallic lithium foil generated by laser ablation is efficiently injected and accelerated by a radio-frequency quadrupole linear accelerator (RFQ linac). We have obtained a peak beam current of 35 mA accelerated to 1.43 MeV, which is two orders of magnitude higher than a conventional injector and accelerator system can deliver.

## Introduction

Neutrons, unlike X-rays or charged particles, have high penetration depth and unique interactions with condensed matter, making them extremely versatile probes for investigating the properties of materials^1–7^. In particular, neutron scattering techniques are often used to study the composition, structure, and internal stress of condensed matter and can give detailed information on minor compounds in metal alloys that are difficult to detect by X-ray spectroscopy^8^. This technique is considered a powerful tool in basic science and has been embraced by metal and other material manufacturers. More recently, neutron diffraction has begun to be applied to detect residual stresses in mechanical components such as rails and aircraft parts^9–12^. Neutrons are also being used in wells to search for oil and gas because they can be easily captured in proton-rich materials^13^. Similar techniques are also being used in the civil engineering field. Non-destructive neutron testing is an effective tool for detecting hidden failures in buildings, tunnels, and bridges. The applications of neutron beams have been actively used in both scientific research and industry, and many of these technologies have historically been developed using nuclear reactors.

However, with the global consensus on nuclear nonproliferation, the construction of small reactors for research purposes is becoming more difficult. Furthermore, the recent Fukushima nuclear accident has made the construction of nuclear reactors almost socially unacceptable. With this trend, the demand for accelerator-driven neutron sources is increasing^2^. Several large accelerator-driven spallation neutron source facilities have already been in operation as an alternative to nuclear reactors^14,15^. However, to utilize the characteristics of the neutron beam more effectively, it is essential to promote the use of compact accelerator-driven sources that can be owned by industrial and university-scale research facilities^16^. An accelerator-driven neutron source adds new functions and features in addition to serving as a replacement for a nuclear reactor^14^. For instance, a linear accelerator-driven generator can easily pulse the neutron flux by manipulating the driver beam. Neutrons are difficult to control once they are emitted, and radiation measurements are difficult to analyze because of the noise caused by background neutrons. Accelerator-driven pulsed neutrons can avoid this problem. Some projects based on proton accelerator technology have been proposed around the world^17–19^. The most popular reactions used in a proton-driven compact neutron generator are ^7^Li(p, n)^7^Be and ^9^Be(p, n)^9^B because they are endothermic reactions^20^. If the energy of the driver proton beam is selected just above the threshold value, excessive radiation and radioactive wastes can be minimized. However, the mass of the target nucleus is much heavier than that of the proton, and the generated neutrons are scattered in all directions. This almost isotropic emission of neutron flux prevents an efficient transport of neutrons to the object under study. Additionally, to produce the required neutron dose at the object position, both the number of driver protons and their energy need to be greatly increased. As a result, a high dose of γ-rays and neutrons will be distributed at large angles and spoil the advantages of the endothermic reactions. A typical proton-based compact accelerator-driven neutron generator has heavy shielding for radiation protection, and it is the most massive part of the system. The necessity of increasing the driver proton energy usually requires an additional increase in the size of the acceleration facility.

**Figure 1:**
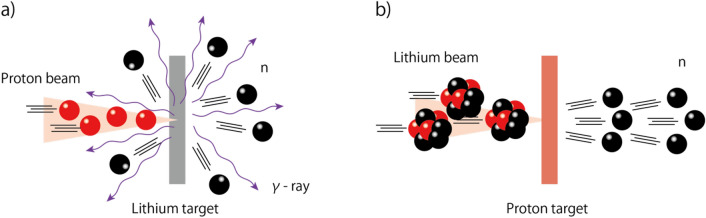
Illustration of neutron production angle for proton versus lithium driver beam (drawn using Adobe illustrator CS5, 15.1.0, https://www.adobe.com/products/illustrator.html). (**a**) Neutrons can be ejected in any direction by this reaction, since the driver proton impinges on a much heavier lithium target atom. (**b**) Conversely, if a lithium-ion driver bombards a hydrogen-rich target, neutrons are generated within a narrow cone in a forward direction, due to the large center of mass velocity of the system.

To overcome the common drawback of conventional accelerator-driven compact neutron sources, an inverse kinematic reaction scheme has been proposed^21^. In this scheme, a heavier lithium-ion beam is used as a driver beam instead of the proton beam, and the target is a hydrogen-rich material such as hydrocarbon plastics, hydrides, hydrogen gas, or hydrogen plasma. An alternative such as a beryllium ion driver beam has been considered; however, beryllium is a toxic material and needs special attention for handling. Hence, lithium is the most suitable beam for the inverse kinematic reaction scheme. Since the momentum of a lithium nucleus is larger than that of a proton, the center of mass of the nuclear collision keeps on moving forward and the neutrons are also emitted in the forward direction. This feature greatly eliminates unwanted γ-rays and neutron emissions at large angles^22^. The comparison of the conventional proton driver case and inverse kinematic scenario is illustrated in Fig. 1.

However, because of the difficulty in producing the necessary flux of high charge state heavy ions compared to protons, only a few neutron generators existed with the inverse kinematic setup^23–25^. All these facilities utilize negative-sputtering ion sources combined with tandem electrostatic accelerators. There are proposals to use other types of ion source to increase the efficiency of beam acceleration^26^. In any case, the available lithium-ion beam current is limited to only 100 μA. There is a proposal to use 1 mA of Li^3+^^27^, but this ion beam current using this method has not been confirmed yet. Intensity-wise, lithium beam-driven accelerators have not been able to compete with proton beam-driven accelerators, which are capable of peak proton currents greater than 10 mA^28^.

To realize a practical compact neutron generator based on a lithium beam, it is favorable to produce high-intensity fully stripped ions. Ions are accelerated and guided by electromagnetic force, and the higher charge state contributes to more efficient acceleration. The lithium-ion beam driver requires more than 10 mA of Li^3+^ peak current.

In this work, we demonstrate Li^3+^ beam acceleration with up to 35 mA peak current, comparable to an advanced proton accelerator. The initial lithium ion beam was created by laser ablation and a direct plasma injection scheme (DPIS), originally developed for C^6+^ acceleration, was applied. A specially designed radio frequency quadrupole linear accelerator (RFQ linac) was fabricated using a four-rod resonating structure^29^. We verified that the accelerated beam had the designed beam energy with high purity. Once the Li^3+^ beam is efficiently captured and accelerated by a radio-frequency (RF) accelerator, a subsequent linear accelerator (linac) section can be used to provide the desired energy to produce an intense neutron flux from the target.

## Results

**Figure 2:**
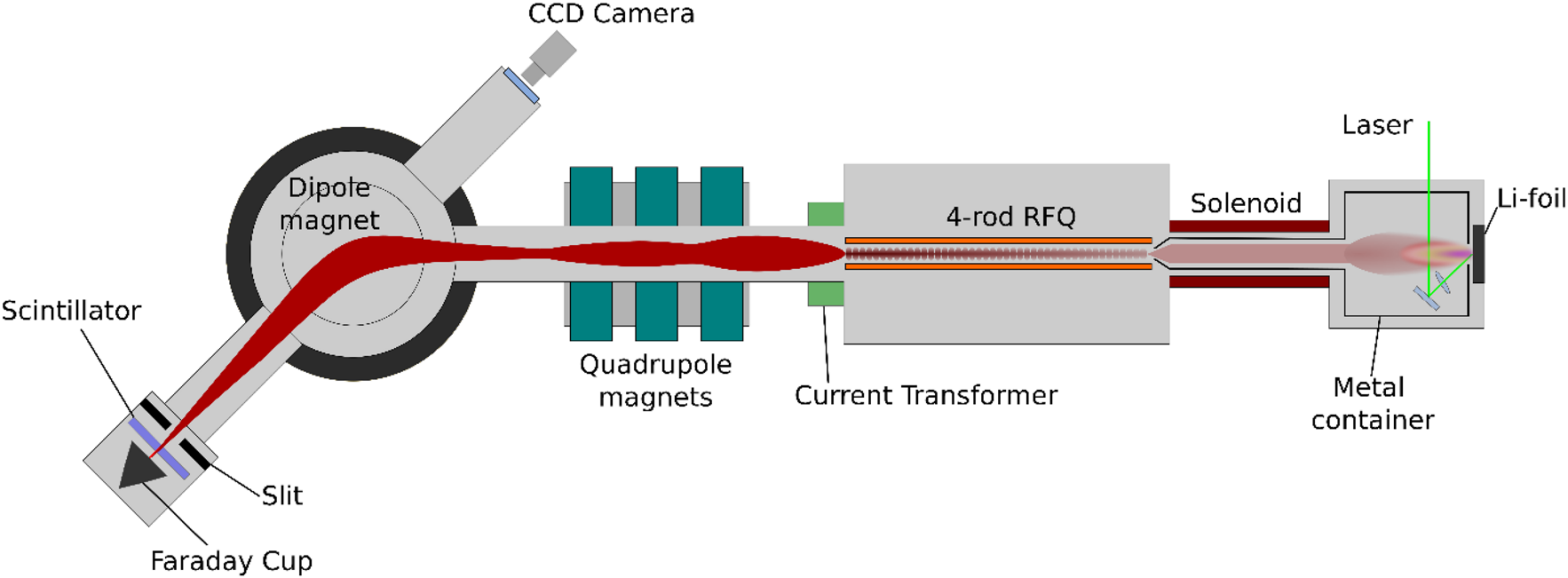
Overview of the experimental apparatus used for beam analysis of the accelerated lithium beams (illustrated by Inkscape, 1.0.2, https://inkscape.org/). From right to left, a laser ablation plasma is produced in the laser-target interaction chamber and is transported toward the RFQ linac. When entering the RFQ linac, the ions are separated from the plasma and injected into the RFQ linac by an abrupt electric field generated by a 52 kV voltage difference between the drift region extraction electrode and the RFQ electrodes. The extracted ions are accelerated from 22 to 204 keV/n while guided by the 2-m-long RFQ electrodes. A current transformer (CT), installed at the exit of the RFQ linac, provides a non-destructive measurement of the ion beam current. The beam is focused by three quadrupole magnets and guided to a dipole magnet that is used to select the Li^3+^ beam and bend it into a detector. Behind a slit, a retractable plastic scintillator and a Faraday cup (FC) biased to -400 V are used to detect the accelerated beam.

The acceleration of high flux ions is a technique that is already well established. The remaining tasks for the realization of the efficient novel compact neutron generator are the generation of a large number of fully stripped lithium ions and the formation of a bunch structure, consisting of a train of ion pulses synchronized with the RF period in the accelerator. The results of experiments designed to achieve this goal will be described in the following three subsections: (1) the generation of the fully stripped lithium ion beam, (2) the acceleration of the beam by a specially designed RFQ linac, and (3) the analysis of the accelerated beam to verify its content. At Brookhaven National Laboratory (BNL), we constructed the experimental setup shown in Fig. 2.

### Generation of fully stripped lithium beam

To produce fully ionized lithium (Li^3+^) ions, it is necessary to generate a plasma with a temperature above their third ionization energy, which is 122.4 eV. We have attempted to generate high-temperature plasmas using laser ablation. This type of laser ion source has not been commonly used to generate lithium-ion beams, since lithium metal is chemically active and requires special handling. We developed a target loading system to minimize contamination caused by humidity and air when the lithium foil was installed into the laser interaction vacuum chamber. All material preparation was performed in a controlled environment of dry argon gas. Once the lithium foil was mounted in the laser target chamber, the foil was irradiated by an Nd:YAG pulsed laser with an energy of 800 mJ per shot. The laser power density was estimated to be about 10^12^ W/cm^2^ at the focal point on the target. When the pulsed laser ablates the target in a vacuum, a plasma is generated. The plasma continues to be heated throughout the 6 ns of laser pulse, mainly by the inverse Bremsstrahlung process. Since no confining external field is applied during the heating stage, the plasma begins to expand three-dimensionally. When the plasma starts to expand at the surface of the target, the center of mass of the plasma gains a velocity perpendicular to the target surface with an energy of 600 eV/n. After heating, the plasma continues to move away from the target axially while expanding isotropically.

As shown in Fig. 2, the ablation plasma expands into a vacuum volume surrounded by a metal container which has the same potential as the target. Therefore, the plasma drifts in the direction of the RFQ linac through an electric field free region. An axial magnetic field is applied between the laser irradiation chamber and the RFQ linac by a solenoid coil wound around the vacuum chamber. The solenoidal magnetic field suppresses the radial expansion of the drifting plasma to preserve the high plasma density during transport to the RFQ aperture. On the other hand, the plasma continues to expand axially during the drift, forming an elongated plasma. A high voltage bias is applied to the metal container enclosing the plasma up to the extraction aperture at the RFQ entrance. The bias voltage is selected to provide the necessary ^7^Li^3+^ injection velocity for proper acceleration by the RFQ linac.

**Figure 3:**
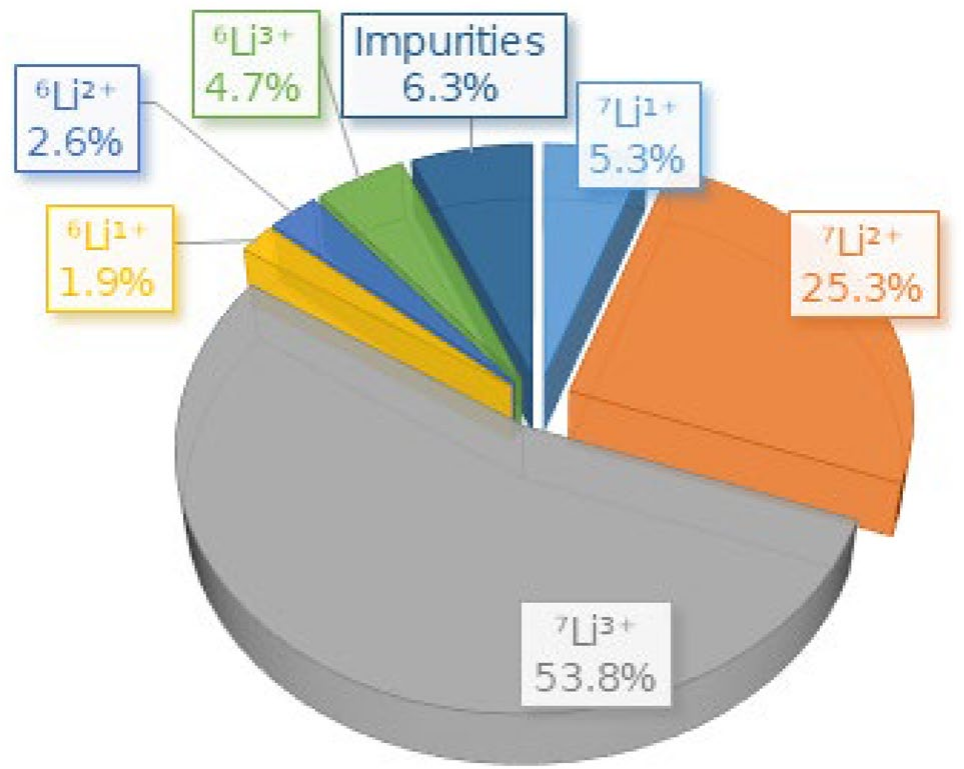
Fraction of ions in the laser-produced plasma obtained using time-of-flight analysis. Ions of ^7^Li^1+^and ^7^Li^2+^ constitute 5% and 25% of the beam, respectively. Within the experimental error, the fraction of ^6^Li species detected is consistent with the natural ^6^Li abundance (7.6%) in the lithium foil target. A small amount of oxygen contamination (6.2%), mainly O^1+^ (2.1%) and O^2+^ (1.5%), was observed, which could be due to surface oxidation of the lithium foil target.

The generated ablation plasma contains not only ^7^Li^3 +^, but also other charge states of lithium and contaminating elements, which are transported simultaneously to the RFQ linac. Prior to the acceleration experiments with the RFQ linac, an off-line time-of-flight (TOF) analysis was performed to investigate the species and energy distributions of the ions in the plasma. The detailed analysis apparatus and observed charge state distribution will be explained in the “Methods” section. The analysis showed that ^7^Li^3+^ ions were the predominant species, which occupy about 54% of the total number of particles as shown in Fig. 3. Based on the analysis, the electric ion current of ^7^Li^3+^ at the ion beam extraction point was estimated to be 1.87 mA. During the acceleration test, a 79 mT solenoid field was applied to the expanding plasma. As a result, the ^7^Li^3+^ current extracted from the plasma and observed at the detector was increased by a factor of thirty.

### Injection and acceleration in RFQ

The lithium plasma drifts in an electric field free region before reaching the RFQ linac as mentioned earlier. At the entrance of the RFQ linac, there is an orifice with a diameter of 6 mm in the metal container, which is biased at 52 kV. The voltage results in an axial acceleration since the electrodes of the RFQ accelerator have zero potential on average, although the voltage of the RFQ electrodes rapidly alternate ± 29 kV at 100 MHz. Because a strong electric field is created at a 10 mm gap between the orifice and the edge of the RFQ electrodes, only positive ions in the plasma are extracted from the plasma at the orifice. In a conventional ion transport system, ions are separated from a plasma by an electric field at a considerable distance before an RFQ linac, and then focused into the RFQ aperture by beam-focusing elements. However, for intense heavy ion beams required for intense neutron sources , a nonlinear repulsion force due to the space charge effect causes significant beam loss in the ion transport system, limiting the peak current that can be accelerated. In our DPIS, high intensity ions are transported as a drifting plasma directly to the extraction point at the RFQ aperture so that ion beam loss does not occur due to space charge. The DPIS was applied to a lithium-ion beam for the first time during the present demonstration.

The RFQ structures were developed to bunch and accelerate low energy high current ion beams and became standard for the first stage of acceleration. We used an RFQ to accelerate ^7^Li^3+^ ions from the injection energy of 22 keV/n to 204 keV/n. Although lower charge state lithium and other species in the plasma were also extracted from the plasma and injected into the RFQ aperture, only ions with a charge to mass ratio (Q/A) close to that of ^7^Li^3+^ could be accelerated by the RFQ linac.

**Figure 4:**
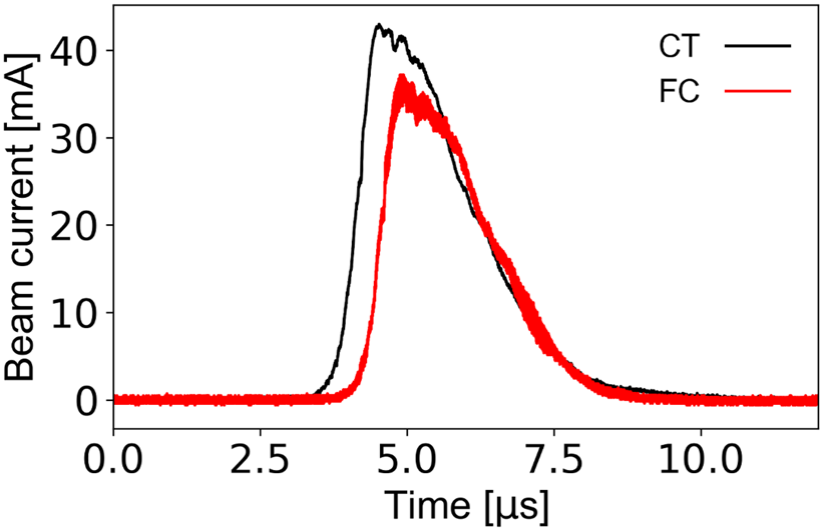
Waveforms of the accelerated beam recorded at the CT (black trace) and the FC (red trace) detector locations. These measurements were triggered by laser light detected by a photodetector when a laser-produced plasma was generated. The black curve shows a waveform measured at the CT attached to the exit of the RFQ linac. Because this detector picks up 100 MHz RF noise due to the proximity of the detector to the RFQ linac, a 98 MHz low pass FFT filter was applied to remove the resonant 100 MHz RF signal imposed on the detected signal. The red curve shows a waveform at the FC after the analyzing magnet guiding the ^7^Li^3+^ ion beam. At this magnetic field, N^6+^ and O^7+^ can be transported in addition to ^7^Li^3+^.

Figure 4 shows the waveforms detected by a current transformer (CT) at the exit of the RFQ linac and by a Faraday cup (FC) after the analyzing magnet, as shown in Fig. 2. The time shift between the waveforms can be explained by the difference of TOF at the detector locations. The measured peak ion current at the CT was 43 mA. At the CT position, the detected beam could contain not only ions that have been accelerated to the designed energy, but also ions other than ^7^Li^3+^ that have not been accelerated sufficiently. However, the similarity of ion current waveforms detected by the CT and the FC implies that the ion current mainly consists of accelerated ^7^Li^3+^ and the decrease of the current peak at the FC was caused by the beam loss during ion transport between the CT and the FC. The beam loss was also confirmed by an envelope simulation. To accurately measure the beam current of ^7^Li^3+^, this beam was analyzed by a dipole magnet as explained in the next section.

### Analysis of accelerated beam

**Figure 5:**
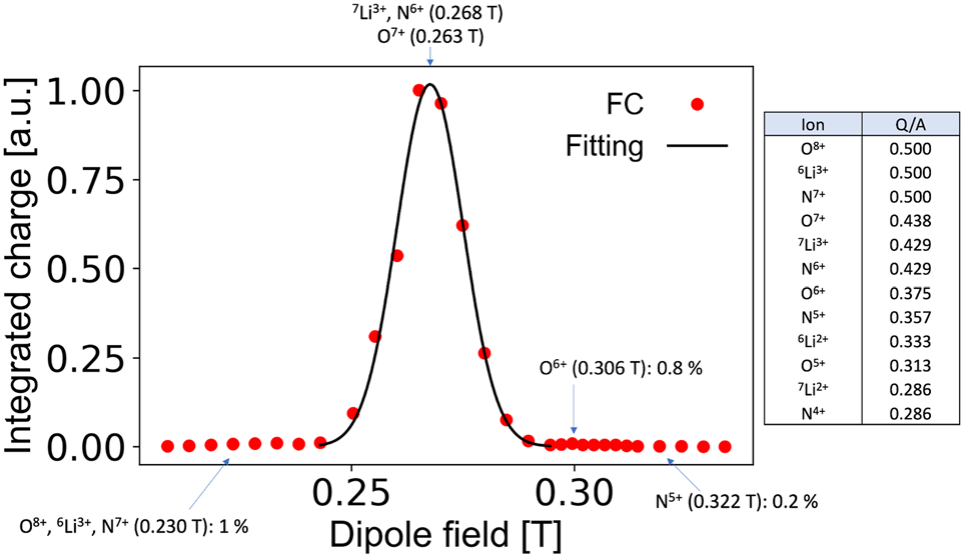
Spectrum of beam constituents obtained by scanning the dipole magnet field. The peak at 0.268 T corresponds to ^7^Li^3+^ and N^6+^. The width of the peak depends on the beam size at the slit. Despite the peak width, ^7^Li^3+^ is well separated from ^6^Li^3+^, O^6+^, and N^5+^ but not from O^7+^ and N^6+^.

The ion beam after the RFQ linac was focused by a series of three quadrupole focusing magnets, then analyzed by a dipole magnet to separate out impurities in the beam. A magnetic field of 0.268 T steered the ^7^Li^3+^ beam to the FC. The detected waveform with this magnetic field is shown by the red curve in Fig. 4. The peak current of the beam reached 35 mA, which is more than 100 times higher than that of a typical Li^3+^ beam obtained in an existing conventional electrostatic accelerator. The beam pulse width was 2.0 μs at the full width at half maximum. The detection of the ^7^Li^3+^ beam with the dipole magnetic field indicates the success of the bunching and the acceleration of the beam. The ion beam current detected by the FC as scanning the dipole magnetic field is shown in Fig. 5. A clean single peak was observed and is well separated from the other peaks. Since all ions accelerated to the design energy by the RFQ linac have the same velocity, beams with similar Q/A are difficult to be separated by a dipole magnetic field. Therefore, we were unable to distinguish ^7^Li^3+^ from N^6+^ or O^7+^. However, the amount of impurities can be estimated from adjacent charge states. For example, N^7+^ and N^5+^ can be easily separated, and N^6+^, which may be part of the impurity, is predicted to be approximately the same amount as N^7+^ and N^5+^. The amount of contamination was estimated to be about 2%.

**Figure 6:**
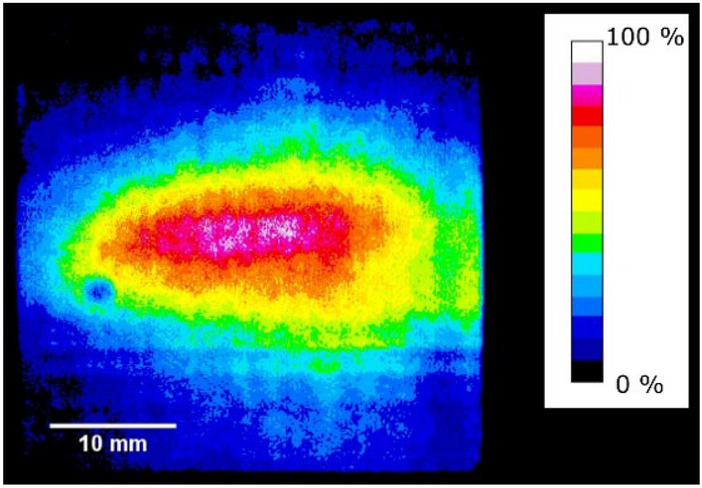
Beam profile observed on a scintillator screen before the FC (colored by Fiji, 2.3.0, https://imagej.net/software/fiji/). The magnetic field of the analyzing dipole magnet was set to guide the Li^3+^ ion beam accelerated to the RFQ design energy. The blue spot in the green area is due to a defective scintillator material.

At the position of the FC, the beam profile was confirmed using an insertable scintillator and recorded by a fast digital camera as shown in Fig. 6. The result indicated that 35 mA of pulsed ^7^Li^3+^ beam was accelerated to the RFQ design energy of 204 keV/n, which corresponds to 1.4 MeV, and transported all the way to the FC detector.

## Discussion

**Figure 7:**
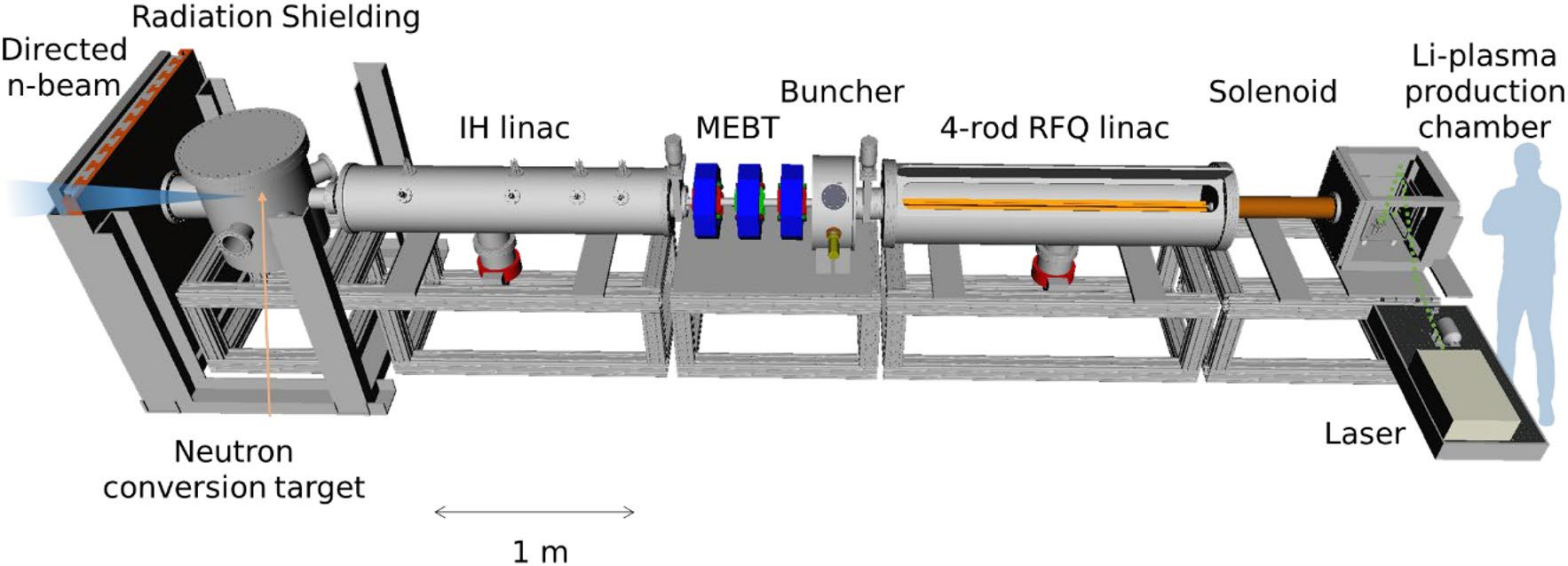
Conceptual design of the proposed compact accelerator-driven neutron source (drawn by Freecad, 0.19, https://www.freecadweb.org/). From right to the left: laser ion source, solenoid magnet, RFQ linac, medium energy beam transport (MEBT), IH linac, and interaction chamber for neutron generation. Since the generated neutron beam is strongly directional, the radiation shielding is provided mainly in the forward direction.

We achieved the production of ^7^Li^3+^ ions by ablating the surface of a solid lithium foil with the laser, and the high-current ion beams were captured and accelerated by the specially designed RFQ linac using the DPIS. The peak ^7^Li^3+^ current achieved at the FC after the analyzing magnet was 35 mA at 1.4 MeV beam energy. This verifies that the most critical part in realizing an inverse kinematics neutron source has been achieved experimentally. In this section of the paper, the entire compact neutron source design, including a high energy accelerator and a neutron target station will be discussed. The design is based on the results obtained with the existing system in our laboratory. It should be noted that the peak ion beam current could be increased further by shortening the distance between the lithium foil and the RFQ linac. Figure 7 illustrates the entire concept of the proposed compact accelerator-driven neutron source.

After a RFQ linac, an Inter-digital H structure linear accelerator (IH linac)^30^ is planned for further acceleration. The IH linac provides a high electric field gradient for the particular velocity range, using a π-mode drift tube structure. A conceptual study was conducted based on a 1D longitudinal dynamics simulation and a 3D envelope simulation. The calculation showed that a 100 MHz IH linac with a reasonable drift tube voltage (less than 450 kV) and focusing magnet strengths can accelerate a 40 mA beam from 1.4 to 14 MeV in 1.8 m. The energy spread at the end of the accelerator chain is estimated to be ± 0.4 MeV, which does not have a significant impact on the energy spectrum of neutrons produced at the neutron conversion target. In addition, the beam emittance is low enough to allow focusing of beams to a smaller beam spot than that of typically required using quadrupole magnets of modest strength and size. In a medium energy beam transport (MEBT) between the RFQ linac and the IH linac, a buncher resonator is used to preserve the bunch structure. To control the beam size in the transverse direction, three quadrupole magnets are used. This design strategy has been used in many accelerator facilities^31–33^. The estimated total length of the entire system from the ion source to the target chamber is less than 8 m, which could be loaded into a standard semi-trailer truck.

The neutron conversion target will be installed immediately downstream from the linear accelerators. We discuss the target station design based on previous studies using inverse kinematics scenarios^23^. The reported conversion targets include solid materials (polypropylene (C_3_H_6_) and titanium hydride (TiH_2_)) and a gas target system. Each target has advantages and disadvantages. Solid targets allow for fine control over thickness. The thinner the target, the more precisely the spatial location of neutron generation is defined. However, such targets may still have some degree of unwanted nuclear reactions and radiation. On the other hand, hydrogen gas targets can achieve a cleaner environment, excluding ^7^Be production, which is the product of the main nuclear reaction. Nevertheless, hydrogen gas has weak stopping power and takes a long physical distance to obtain enough energy deposition. This is slightly disadvantageous for TOF measurements. Additionally, if a thin film is used to seal the hydrogen gas target, the production of γ-rays from the film and energy loss of the incident lithium beam must be taken into account.

A polypropylene target had been used at LICORNE and the target system was updated to a hydrogen gas cell sealed by a tantalum foil. Both target systems can produce up to 10^7^ n/s/sr, assuming a beam current of 100 nA for ^7^Li^34^. If we apply this reported neutron yield conversion to our proposed neutron source, 7 × 10^–8^ C of the lithium driver beam can be delivered for each laser shot. This means that only two laser shots per second will provide 40% more neutrons than that LICORNE provides in a continuous beam for one second. When the frequency of laser shots is increased, the total flux can easily increase. If we assume a 1 kHz laser system, which is available in the market, the average neutron flux can be simply scaled to about 7 × 10^9^ n/s/sr.

When we employ a high repetition rate of the system with a plastic target, heat deposition on the target should be controlled, since polypropylene, for example, has a low melting point of 145—175 °C and a low thermal conductivity of 0.1—0.22 W/m/K. For 14 MeV lithium-ion beams, a 7 μm thick polypropylene target is enough to reduce the beam energy down to the reaction threshold (13.098 MeV). Considering the total impact of ions produced by a single laser shot to the target, the energy deposition of the lithium-ions passing through the polypropylene is estimated to be 64 mJ/pulse. Assuming that all the energy is transferred within a circle 10 mm in diameter, the corresponding temperature rise per pulse is about 18 K/pulse. The energy deposition on the polypropylene target is based on the simple assumption that all the energy loss is accumulated as heat, without radiation loss or other heat loss. Since increasing the number of pulses per second requires removal of heat buildup, we may use a tape-shaped target to avoid energy deposition at the same spot^23^. Assuming a 10 mm beam spot on the target with a 100 Hz laser repetition rate, the scanning speed of the tape polypropylene will be 1 m/s. If overlapping of the beam spots is allowed, a higher repetition rate is feasible.

We also investigated a hydrogen gas cell target, because a more intense driver beam can be used without target damage. The neutron beam can be easily adjusted by varying the length of the gas cell and the pressure of the hydrogen gas inside. To separate the target gas region and the vacuum in the accelerator, a thin metal foil is typically used. Accordingly, the incident lithium beam energy should be increased to compensate for the energy loss at the foil. The described target assembly in the report^35^ consists of a 3.5-cm-long aluminum container with an H_2_ gas pressure of 1.5 atm. The 16.75 MeV lithium beam enters the cell through an air-cooled 2.7 μm-thick Ta foil and the energy of the lithium beam at the end of the cell decelerates to the reaction threshold. To increase the lithium beam energy from 14.0 MeV to 16.75 MeV, the IH linac has to be extended by about 30 cm.

Neutron emission from the gas cell target was also investigated. For the gas target described above at LICORNE, GEANT4^36^ simulation shows that highly directed neutrons are produced within a cone as shown in Fig. 1 in Ref.^37^. Reference^35^ shows that the energy range was between 0.7 and 3.0 MeV and the maximum cone opening was 19.5° with respect to the propagation direction of the primary beam. The highly directed neutrons allow a significant reduction in the amount of shielding material at most angles, reducing the weight of the structure and providing more flexibility when installing the measuring devices. Regarding radiation protection, besides neutrons, such a gas target system also isotropically emits 478 keV γ-rays in the center of mass frame^38^. These γ-rays are produced from the ^7^Be decay and by the deexcitation of ^7^Li which is produced when the primary Li beam hits the Ta entrance window. However, a strong reduction of the background is achieved by adding a thick Pb/Cu cylindrical collimator^35^.

As an alternative target, we can employ a plasma window^39,40^ that allows us to achieve relatively high hydrogen pressure and a small spatial area for neutron generation, although it is still not as good as a solid target.

We are exploring the options of neutron conversion targets for the expected energy distribution and beam size of our lithium beam using GEANT4. Our simulations show consistent neutron energy and angular distribution for the hydrogen gas target shown above in the literature. In any target systems, highly directed neutrons can be generated by inverse kinematic reaction driven by intense ^7^Li^3+^ beam on a hydrogen-rich target. Thus, a new neutron source can be realized by combining already established techniques.

## Methods

### Off-line time-of-flight (TOF) laser plasma analysis

The laser irradiation conditions replicated the ion-beam production experiments before the acceleration demonstration. The laser was a tabletop nanosecond Nd:YAG system employed at a laser power density of 10^12^ W/cm^2^ at a fundamental wavelength of 1064 nm, spot energy of 800 mJ, and 6 ns per pulse duration. The spot size on the target was estimated to be 100 μm in diameter. Since metallic lithium (Alfa Aesar, 99.9% purity) is sufficiently soft, the precisely cut material was pressed into the mold. The dimensions of the foil were 25 mm × 25 mm with a thickness of 0.6 mm. When the laser was irradiated, crater-like damage occurred on the target surface, so the target was moved by a motorized stage to provide a fresh part of the target surface for each laser shot. To avoid recombination due to residual gas, the pressure in the chamber was kept below the 10^–4^ Pa range.

The initial laser plasma has a small volume since the laser spot size was 100 μm and within 6 ns from its creation. It can be assumed that the volume is a pinpoint and then expands. If we put a detector at a certain distance *x* m from the target surface, the signal obtained follows the relations below for the ion current *I*, the arrival time of the ions *t*, and the pulse width *τ*.

1 $$ I \propto 1/x^{3} $$

2 $$ t \propto x $$

3 $$ \tau \propto x $$

The generated plasmas were examined by TOF through the FC and energy ion analyzer (EIA) located 2.4 m and 3.85 m away from the laser target. The FC had a suppressor mesh biased at -5 kV to prevent electrons. The EIA had a 90-degree electrostatic deflector, which consists of two coaxial metal cylinder electrodes, with the same voltage but with opposing polarity, the outer positive and the inner negative. The expanding plasma was guided to the deflector behind a slit and bent by the electric field across the cylinders. An ion that satisfies the relation *E/z* = *eKU* is detected by the secondary electron multiplier (SEM) (Hamamatsu R2362), where *E*, *z*, *e*, *K*, and *U* are the energy of the ion, the charge state, the charge of the electron, the EIA geometry factor and the potential difference between the electrodes, respectively. When the voltage across the deflector was varied, the energy and charge-state distribution of the ions in the plasma was obtained. The scanning voltage, *U*/2, of the EIA ranges from 0.2 V to 800 V, corresponding to ion energies of 4 eV to 16 keV per charge state.

**Figure 8:**
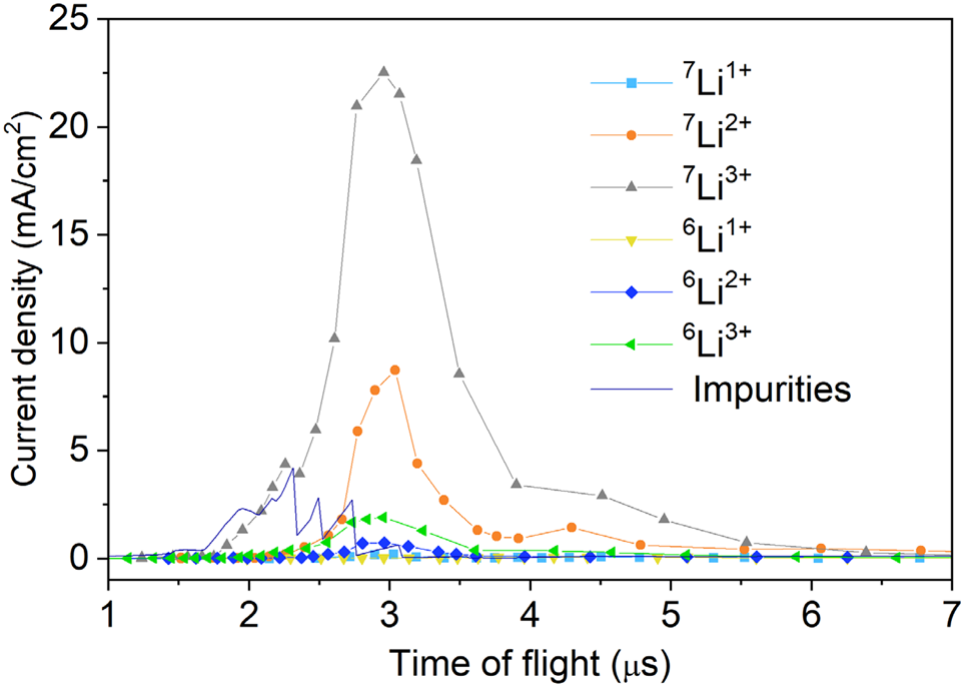
Analyzed ion charge state distribution. This is a temporal profile of ion current density analyzed by an EIA and scaled at 1 m away from a lithium foil using Eq. (1) and (2). The laser irradiation condition described in the section “Generation of fully stripped lithium beam” was used. By integrating each current density, the fractions of ions in the plasma shown in Fig. 3 were calculated.

The analyzed ion charge state distribution with the laser irradiation condition described in the section “Generation of fully stripped lithium beam” is shown in Fig. 8.

### Ion source

A laser ion source can provide multi-milliampere class intense ion beams with high charge states. However, it has not been used commonly since the beam delivery was very difficult due to space charge repulsion force. In the conventional scheme, an ion beam is extracted from the plasma and transported to the first stage accelerator through a beamline, which has some focusing magnets to shape the ion beam to match the acceptance of the accelerator. In the beamline, the space charge force non-linearly diverges the beam, especially in the low-velocity region, and serious beam losses are observed. To overcome this issue in the design of medical carbon accelerators, a new beam delivery scheme has been proposed, i.e., the DPIS^41^. We have applied this technique to accelerate the intense lithium-ion beam for the new neutron source.

The space, where the plasma was generated and expanded, was surrounded by a metal container as seen in Fig. 4. The enclosed space was extended to the entrance of the RFQ resonator, including the volume inside the solenoid coil. A voltage of 52 kV was applied to the container. In the RFQ resonator, the ions were extracted by the potential through a 6 mm diameter aperture, because the RFQ is grounded. The non-linear repulsive forces at the beamline can be eliminated since ions are delivered with a plasma state. In addition, as mentioned above, we applied a solenoid field combined with the DPIS to control and enhance the ion density at the extraction orifice.

### Design of RFQ linear accelerator

**Figure 9:**
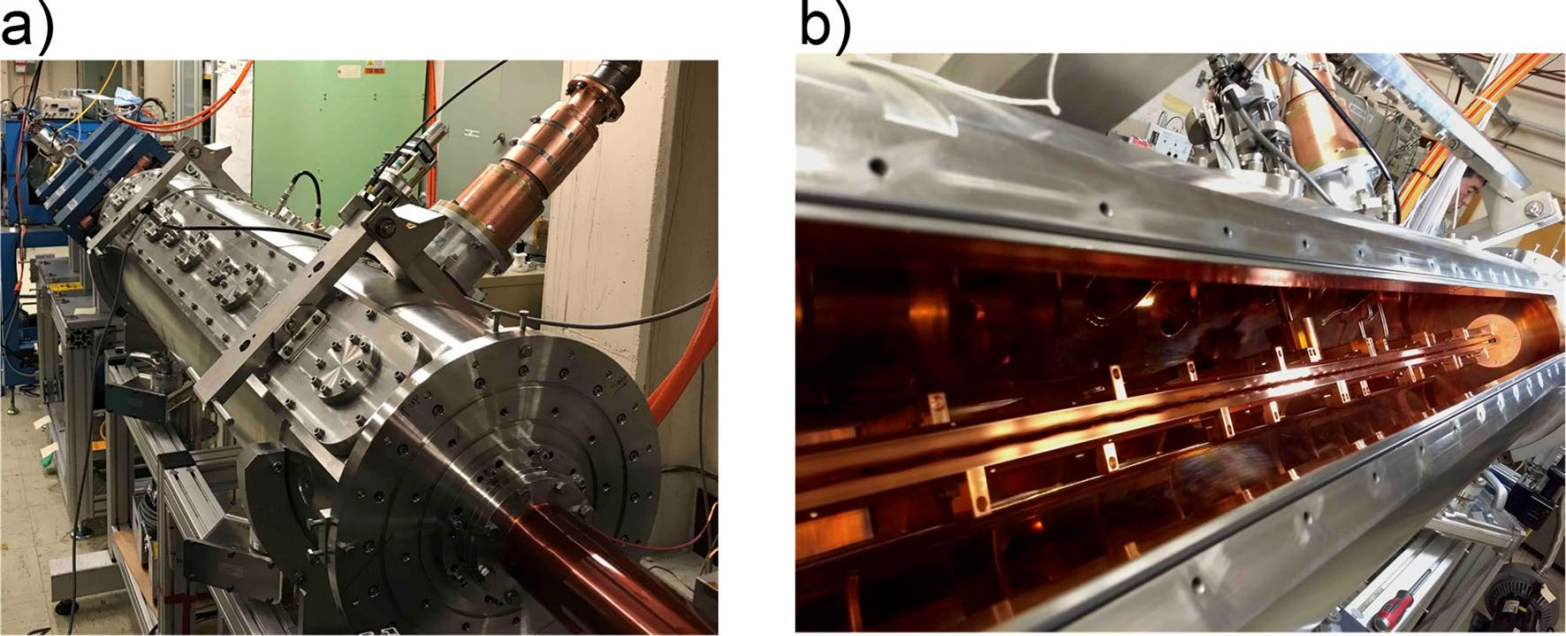
RFQ linac accelerator. (**a**) (left) The outside of the RFQ linac chamber. (**b**) (right) The 4-rod electrodes in the chamber.

The RFQ linac is composed of a cylindrical vacuum chamber as shown in Fig. 9a. Inside of it, four oxygen-free copper rods are placed with quadrupole symmetry around a beam axis (Fig. 9b). The 4 rods and the chamber form a resonant RF circuit. The induced RF field produces a time-varying voltage on the rods. Ions injected around the axis in the longitudinal direction are confined transversely by the quadrupole field. Meanwhile, the tips of the rods are modulated to generate an axial electric field. The axial field divides the injected continuous beam into a series of beam pulses, called beam bunches. Each bunch is contained in a certain time duration within one RF period (10 ns). The adjacent bunches are spaced according to the RF period. In the RFQ linac, the 2 μs beam from the laser ion source is transformed into a train of 200 beam bunches. The bunches are then accelerated to the designed energy.

The main design parameters for the RFQ linac are the rod voltage, the resonant frequency, the beam bore radius, and the modulation of the electrodes. The rod voltage, ± 29 kV, was selected to have an electric field lower than a threshold for electrical breakdown. With a lower resonant frequency, the transverse focusing force is larger, while the averaged acceleration field is smaller. A larger bore radius can accept a larger beam size and hence a larger beam current due to smaller space charge repulsion force. On the other hand, a larger bore radius requires larger RF power to energize the RFQ linac. In addition, it is limited from the field quality requirement. From these balances, the resonant frequency (100 MHz) and the bore radius (4.5 mm) were selected for the high current beam acceleration. The modulation was chosen to make beam bunches with small losses and maximize the acceleration efficiency. The design is iteratively optimized and a RFQ linac design was obtained that accelerates 40 mA of ^7^Li^3+^ ions from 22 to 204 keV/n in 2 m. The RF power was 77 kW, measured during the experiment.

### Beam analyzer

An RFQ linac can accelerate ions that have a certain range of Q/A. Therefore, isotopes and other species must be taken into account to analyze beams transported to the end of the linac. In addition, the desired ions that are partially accelerated but dropped from the acceleration condition in the middle of the accelerator can still satisfy the transverse confinement and can be delivered to the end. The undesired beams other than design particles of ^7^Li^3+^ are called impurities. In our experiment, the main concerns about impurities are ^14^N^6+^ and ^16^O^7+^ because lithium metal foil can react with oxygen and nitrogen in the air. These ions have a Q/A that can be accelerated together with ^7^Li^3+^. We used a dipole magnet to separate beams of different Q/A to analyze beams after the RFQ linac.

The beamline after the RFQ linac was designed to transport fully accelerated ^7^Li^3+^ beams toward the FC after the dipole magnet. An electrode biased at − 400 V was used to suppress secondary electrons from the cup to accurately measure the ion beam current. With these optics, the ion trajectories were separated in the dipole and focused on different positions depending on the Q/A. The beam at the focal position has a certain width due to several factors, such as momentum spread and space charge repulsion force. Only when the distance between focal positions of two ion species is larger than the beam width, the species can be separated. To achieve as much resolution as possible, horizontal slits were installed near the waist of the beam, where the beams are nearly focused. There was a scintillator screen (CsI (Tl) from Saint-Gobain, 40 mm × 40 mm × 3 mm) between the slits and the FC. The scintillator was used to determine the minimum slit size for design particles to just pass through to achieve the best resolution, and to demonstrate that reasonable beam size of the high current heavy ion beam can be realized. The beam image on the scintillator was taken by a CCD camera through a vacuum window. The exposure time window was adjusted to cover the entire pulse width of the beam.

## Data Availability

The datasets used or analyzed during the current study are available from the corresponding author on reasonable request.
